# Ultra Small Integrated Optical Fiber Sensing System

**DOI:** 10.3390/s120912052

**Published:** 2012-09-03

**Authors:** Bram Van Hoe, Graham Lee, Erwin Bosman, Jeroen Missinne, Sandeep Kalathimekkad, Oliver Maskery, David J. Webb, Kate Sugden, Peter Van Daele, Geert Van Steenberge

**Affiliations:** 1 Centre for Microsystems Technology (CMST), Ghent University - IMEC, Elis Department, Technologiepark 914A, B-9052 Zwijnaarde, Belgium; E-Mails: erwin.bosman@elis.ugent.be (E.B.); jeroen.missinne@elis.ugent.be (J.M.); sandeep.kalathimekkad@ugent.be (S.K.); geert.vansteenberge@elis.ugent.be (G.V.S.); 2 Aston Institute of Photonic Technologies, Aston University, Aston Triangle, Birmingham B4 7ET, UK; E-Mails: leegcb@aston.ac.uk (G.L.); maskeryo@aston.ac.uk (O.M.); d.j.webb@aston.ac.uk (D.J.W.); k.sugden@aston.ac.uk (K.S.); 3 Centre for Microsystems Technology (CMST), Ghent University - IMEC, Intec Department, Technologiepark 914A, B-9052 Zwijnaarde, Belgium; E-Mail: peter.vandaele@intec.ugent.be

**Keywords:** dynamic read-out, fiber sensor, flexible, integration, low-cost, polymer embedding, ultra thin optoelectronic chip package, VCSEL

## Abstract

This paper introduces a revolutionary way to interrogate optical fiber sensors based on fiber Bragg gratings (FBGs) and to integrate the necessary driving optoelectronic components with the sensor elements. Low-cost optoelectronic chips are used to interrogate the optical fibers, creating a portable dynamic sensing system as an alternative for the traditionally bulky and expensive fiber sensor interrogation units. The possibility to embed these laser and detector chips is demonstrated resulting in an ultra thin flexible optoelectronic package of only 40 μm, provided with an integrated planar fiber pigtail. The result is a fully embedded flexible sensing system with a thickness of only 1 mm, based on a single Vertical-Cavity Surface-Emitting Laser (VCSEL), fiber sensor and photodetector chip. Temperature, strain and electrodynamic shaking tests have been performed on our system, not limited to static read-out measurements but dynamically reconstructing full spectral information datasets.

## 1. Introduction

Traditional sensors for measuring physical quantities such as temperature, strain or pressure are mostly based on electrical working principles such as changing resistance or capacitance. The main drawbacks of these sensors are related to the sensitivity, cross talk, non-compatibility with harsh environments and their size. Although several alternatives based on MicroElectroMechanical Systems (MEMS) technology have been developed and are available on the market [1], a major market share is not yet addressed by these traditional electrical sensors.

Optical alternatives are becoming increasingly important as the environments in which sensors are being deployed are becoming more and more challenging. Structural health monitoring applications such as stress monitoring in bridges, air plane wings or wind turbines, as well as biomedical applications such as heart beat and respiration monitoring or even pressure measurements in the esophagus of patients, require sensors which are small, lightweight, chemically inert and preferably electrically isolated. Optical fiber sensors, based on fiber Bragg gratings reflecting particular wavelengths of light, can meet these needs and offer additional advantages such as easy multiplexing, mechanical flexibility and a low-cost sensor base material [2,3].

However, using optical fiber sensors requires a spectral read-out unit, as the sensing information is encoded in the shift, deformation or split of the optical Bragg wavelength. Commercially available types of spectral read-out units are mostly based on a broadband light source, for example a superluminescent light-emitting diode (LED), spectrometer or reference grating, and are typically bulky (therefore not mechanically flexible) and expensive. We present a unique fiber sensor interrogation system, based on electro-thermal wavelength tuning of an optoelectronic source, which can be fabricated low-cost and with an ultra small form factor. By using simple optoelectronic components (VCSEL and photodetector) and dedicated fiber coupling techniques, an ultra thin optical chip package is fabricated to embed laser and detector chips in flexible polymer foils with a thickness of only 40 μm. An integrated fiber coupling technique, based on a 45° micromirror, is used to ensure compatibility of the pigtailed optoelectronic components with planar flexible sensing sheets of only 1 mm thick, incorporating both driving and read-out optoelectronics as well as the optical sensor elements.

This paper describes a proof of concept set-up in which the fiber sensor system is designed, tested and characterized. A respiration monitoring system, with wireless data link and Android read-out application, is built as an example. Then, the technology to embed and pigtail the driving optoelectronic components in ultra thin packages is discussed. Finally, temperature, axial strain and tactile sensing tests are performed on the fully embedded system. Full reconstruction of the spectral response of the Bragg grating, with a spectral acquisition speed up to 1 kHz, is demonstrated using these embedded components, consequently avoiding bulky and expensive read-out units.

## 2. Sensor and Read-Out Principle

### 2.1. Fiber Bragg Grating Sensors

A fiber Bragg grating is a distributed mirror in a short segment of the optical fiber, reflecting a limited wavelength range and transmitting all others. The working principle is based on a periodic variation of the refractive index of the fiber core. Optical fiber sensors are fabricated from low-cost base materials, typically silica, and can be deployed in harsh conditions such as an explosive environment, high pressure gas applications, air plane wings or wind turbines, as well as in medical treatments which often do not accommodate active electronic components. Recently, polymer optical fiber sensors have also been developed based on polymethyl methacrylate (PMMA) [4], possessing additional advantages such as inherent biocompatibility and enhanced sensitivity. The typical fabrication process of an optical fiber sensor consists of different process steps, including fiber drawing, fiber coating and Bragg grating inscription. Inscribing a Bragg grating is mostly achieved by using an interference pattern of a UV (e.g., excimer) laser source created by 2-beam interference or phase masks [5]. Alternatively, direct writing by a femtosecond laser can be used [6]. The resulting periodic refractive index variation is acting as a wavelength-selective filter with a central reflecting wavelength *λ_c_*. The sensing information is encoded in the optical spectrum which is reflected by the fiber Bragg grating. An external perturbation (temperature, strain or pressure variation) causes the central Bragg wavelength *λ_c_* to shift.

**Figure 1.**
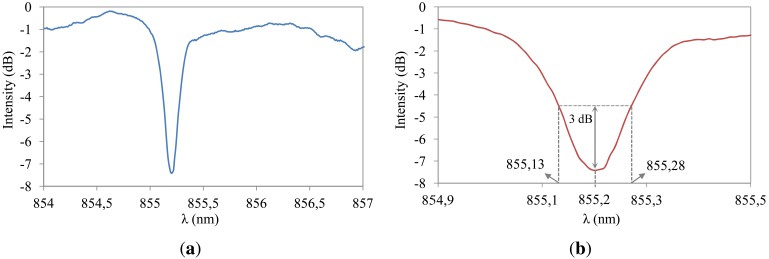
Typical spectral response of the fabricated fiber Bragg gratings at 850 nm, measured in transmission with an optical spectrum analyzer. (**a**) Normalized transmission spectrum of the grating; (**b**) Detailed view indicating the 3 dB wavelength range in transmission.

The FBG sensors for this work were inscribed in Fibercore SM800, a B/Ge co-doped optical silica fiber, with a 244 nm UV laser using a holographic technique [7]. Prior to UV inscription, fibers were submitted to hydrogenation at 200 bar and 80 °C for 48 hours and stored for a short period at −40 °C. This fiber type has a very high photosensitivity to enable rapid fabrication of gratings. The gratings were fabricated with a center wavelength at 855 nm to match the wavelength tuning range of the VCSEL devices used to drive the fiber sensors. A typical FBG transmission spectrum fabricated using this type of fiber is illustrated in Figure 1. The 3 dB wavelength range, measured in transmission as depicted in Figure 1(b), is 0.15 nm. A broadband source (superluminescent LED) and an optical spectrum analyzer were used to perform these initial spectral measurements. The main advantage of using fiber sensors at 850 nm is the availability of low-cost semiconductor laser and detector chips, which is exploited in this paper by the use of these silica fiber Bragg gratings in combination with small and low-cost read-out units.

### 2.2. Interrogation Principle

**Figure 2.**
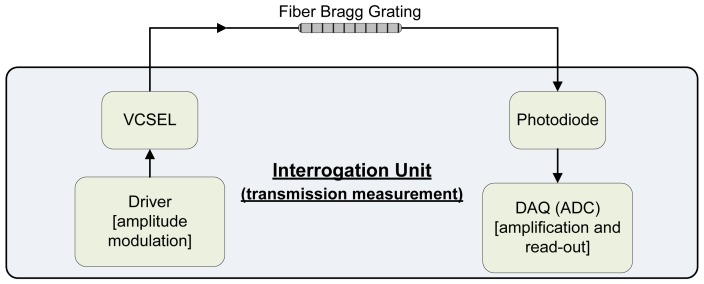
Schematic view of the sensor interrogation system.

The interrogation scheme we present is schematically shown in Figure 2. A single-mode VCSEL is used as a fiber optic driving unit and a photodiode is used to read out the transmitted power. The thermoelectric effect causes the emitting wavelength to shift when tuning the VCSEL driving current. Higher driving currents correspond to higher redshifts in the VCSEL wavelength. An electrical driving unit is needed to modulate the electrical current in the VCSEL, consequently activating the VCSEL wavelength redshifting. Depending on the separation of the different Bragg gratings, this interrogation system even has limited possibility to read out multiplexed fiber sensing points. A data acquisition board linked to a computer is typically used to store and visualize the photocurrent through the photodiode.

Fiber sensing systems based on a similar VCSEL wavelength redshifting interrogation technique have been reported by other institutes and include vibration [8] and temperature [9] sensing systems. Using MEMS technology, it is possible to extend the tuning range of the VCSEL increasing the number of addressable multiplexed sensing points [10,11]. Other types of laser diodes can also be used to perform wavelength tuning enabling fiber sensor interrogation [12]. Alternatively, tunable optical filters have been applied on broadband light sources or detectors, such as a driving superluminescent LED [13] or a read-out photodetector [14].

**Figure 3.**
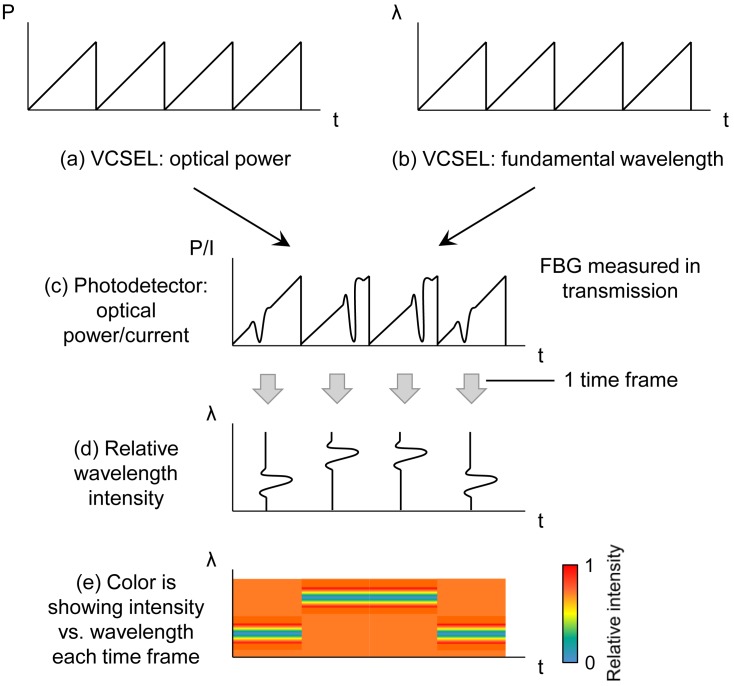
Schematic example of the fiber sensor interrogation principle *(modulation mode)*.

The interrogation system has two operating modes: modulation mode and constant current mode. Figure 3 shows a basic schematic example of the dynamic interrogation scheme *(modulation mode)*. In Figure 3(a,b), the VCSEL is modulated with a sawtooth signal and the optical power and wavelength are varying accordingly. Figure 3(c) shows the response of the photodetector current when a Bragg grating is measured in transmission. The photocurrent is depicting the sawtooth driving signal and the grating filter characteristic. Combining the data in Figure 3(c) with the data from a VCSEL calibration measurement enables filtering the sawtooth signal and isolating the grating response in the photocurrent signal (Figure 3(d)). Assuming the VCSEL is modulated using a frequency f, a new full spectrum reconstruction is available each 1/f seconds. Assigning a color to the relative wavelength intensity results in a complete spectral reconstruction (Figure 3(e)) in which the intensity is depicted for each wavelength, every 1/f seconds.

Alternatively, a fixed VCSEL driving current can be used. The varying transmitted optical power intensity is then monitored at a well-defined wavelength *(constant current mode)*. This wavelength is preferably on the edge of the grating characteristic, enabling the distinction between blue- and redshifting of the fiber sensor. In this static interrogation scheme, one is limited to the detection of the amount of Bragg wavelength shift and consequently losing the dynamic sensing information such as peak broadening, splitting or deformation.

The remainder of this paper is divided in two parts: the first part is exploiting the interrogation system to come up with a low-cost, portable system and the second part is taking the technology one step further by integrating the opto-electrical components in thin, flexible packages and consequently limiting the thickness of the total sensing system to one millimeter.

## 3. Proof of Concept: Illustration of the Sensor System Using Conventional Packaged Optoelectronic Components

**Figure 4.**
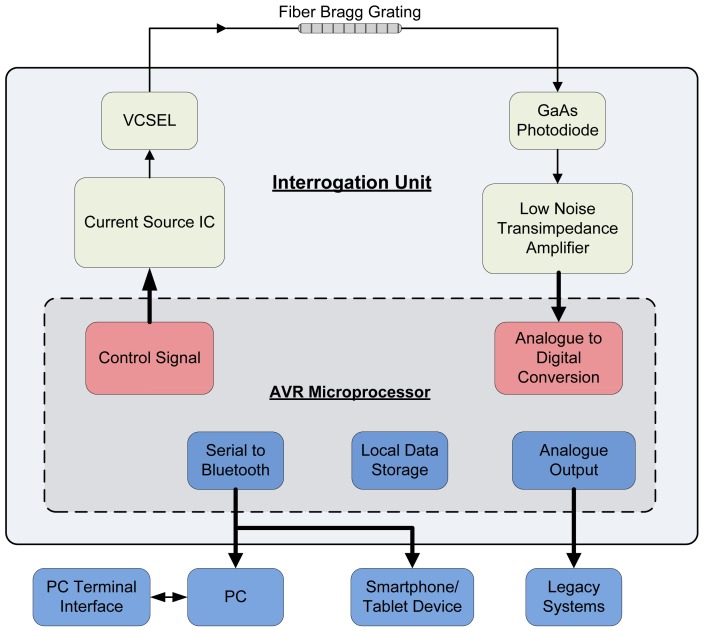
Detailed schematic view of the proof of concept sensor interrogation system.

To demonstrate the capabilities of the interrogation principle, an FBG was set up in transmission mode using a VCSEL light source and a GaAs photodetector as shown in Figure 4. Note that it is also possible to configure a grating in reflection mode by adding a coupler. This enables higher signal to noise ratio measurements of the grating but with the trade off in having to include a coupler.

### 3.1. Inside the Interrogation Unit

**Figure 5.**
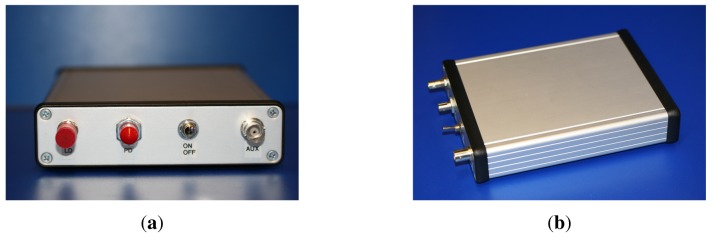
Interrogation system incorporating all elements schematically shown in Figure 4. (**a**) Front panel view. Ports from left-to-right: laser diode (fiber out), photodetector (fiber in), on/off switch, analogue auxiliary port; (**b**) Side view. Power inlet port is situated at the rear.

The interrogation unit is shown in Figure 5, has a dimension of 160 mm × 116 mm × 35 mm and weighs 520 g with a lithium-ion battery. The unit can run up to 24 hours on a single charge or indefinitely when plugged into a power outlet. The interrogation unit was built around an Atmel ATMEGA644 microcontroller running a custom written program. The Atmel ATMEGA family of microcontrollers were chosen for their low-power consumption, low-cost and high-performance. The Atmel ATMEGA644 microcontroller features 64 KB ISP flash memory, 2 KB EEPROM, 4 KB SRAM, selectable 8 or 10-bit analogue to digital converters (ADC), 2 USARTs and 32 general purpose I/O channels. The primary method used to control and configure the interrogation unit is through a graphical user interface in the form of an application (app) built on the Android platform which communicates wirelessly over Bluetooth to a tablet or smartphone device. The system can also be set up using a computer terminal using the serial RS232/Bluetooth connection.

The microcontroller directly controls a current source (Wavelength Electronics LDD P series) which was used to drive a VCSEL (or other light source). The photodetector was connected through a low noise transimpedance amplifier with a variable gain which converts the photocurrent into a voltage signal which is then read by the on-board microcontroller ADC. The variable gain in the transimpedance amplifier offers additional flexibility to allow the system to be fine-tuned and adapted to suit different strength gratings and light sources.

As discussed in the previous section, the interrogation unit has two modes of operation—constant current mode and modulation mode. Under constant current mode, the interrogation unit sweeps the drive current of the VCSEL using a user configured step size to reconstruct the profile of the FBG. Once the grating profile has been established, the system automatically chooses the VCSEL driving current which matches the source wavelength with the most sensitivity edge of the FBG. With a static drive current, the interrogation unit then monitors the amplitude of the light received by the photodetector. The system can sample the photodetector up to a rate of 5 kHz; moving point averages and other math calculations can be computed directly on the microcontroller. In modulation mode, the Android app is used to select a modulation signal such as a sine or sawtooth signal to drive the current source. The microcontroller is able to modulate the current source at a frequency up to 1 kHz. A higher sensing sensitivity can be achieved in modulation mode due to the higher signal to noise ratio and therefore much smaller perturbations on the FBG can be detected compared to constant current mode. Initial testing of the electronics in combination with a fiber sensor element can be found in [15].

### 3.2. Read-Out Android App

**Figure 6.**
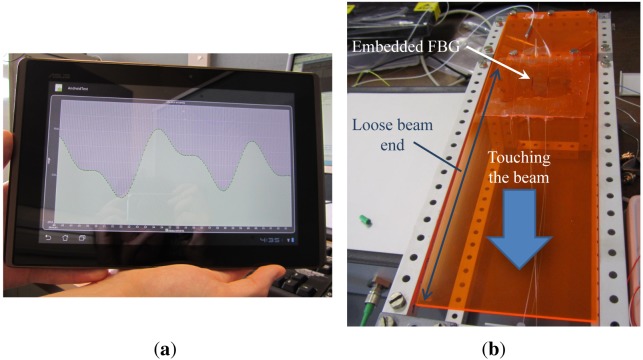
Proof of concept characterization set-up. (**a**) Android read-out system; (**b**) Beam test set-up with one end free to oscillate.

The Android app (Figure 6(a)) used to configure the interrogation unit also acts as a data logger and live data plotter for perturbations experienced by the FBG. It is also possible to recall data plots collected in past sessions. Alternatively, data can be logged and plotted through a PC terminal connected through the RS232/Bluetooth serial interface. The Android device automatically connects wirelessly over Bluetooth to the interrogation unit on startup. When a connection has been established, a prompt is displayed to allow the user to select the mode of operation, configure the maximum drive current limit, modulation type and modulation frequency. Once the settings are saved, a new measurement session is displayed in the form of a live graph with the photodetector intensity plotted against a data reading identifier code. Incoming data is automatically logged and saved to the internal memory of the tablet or smartphone device which can later be retrieved and replotted. In addition, the 2 KB EEPROM microcontroller on-board memory can provide ample space to store up to 500 data points. The analogue auxiliary output port can be used simultaneously with the Android app and the on-board memory. The analogue port provides a quick and easy to use interface to connect to an oscilloscope or other legacy equipment to view live data at high modulation rates.

### 3.3. Proof of Concept Demonstration

**Figure 7.**
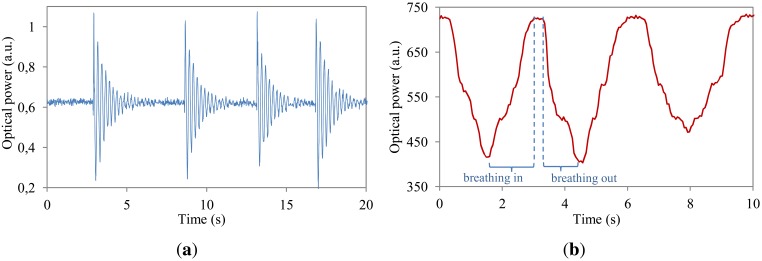
Proof of concept characterization results. (**a**) Transmitted power oscillations resulting from a downward touching force at 2.9 s, 8.7 s, 13.2 s and 16.9 s; (**b**) Breathing movement system response.

The interrogation unit was tested with a commercially available pigtailed VCSEL and photodetector from Honeywell in the interrogation box. An FBG was used as the sensor device which was embedded in a flexible and stretchable polymer host material and taped to a beam secured at one end so that the beam was free to oscillate when a downward force was applied (Figure 6(b)). The polymer host material ensures compatibility with irregularly shaped objects such as robots or the human body. For the first test, the interrogation unit was set to constant current mode and the microcontroller swept the VCSEL drive current from 0.1 mA to 4 mA with a step size of 0.01 mA—which corresponded to a wavelength tuning range of 855.877 nm to 857.660 nm. During the sweep, the photodetector was sampled at 5 kHz with every 50 readings averaged and plotted onto a real time graph on the Android device. Once the sweep completed, the microcontroller automatically selected the VCSEL drive current that corresponded to the most sensitive edge of the FBG. After the set-up sequence, the beam with the grating was made to oscillate to demonstrate the speed and type of read-out that is possible with the interrogation unit. A screen capture of the live graph (transmitted optical power) is shown in Figure 7(a). Both blue- (decreasing optical power) and red-shifting (increasing optical power) effects of the FBG are visible. Figure 7(b) shows the system response when mimicking breathing movements on the embedded fiber. Next to the constant current mode, also the modulation mode was successfully tested using similar driving parameters but with continuous current sweeping.

## 4. Towards an Ultra Small Fully Embedded Sensor System

To come up with a flexible and fully integrated fiber sensor system, a dedicated optoelectronic chip package is needed to replace the conventional optoelectronic packages (for example Transmitter Optical Sub-Assembly or butterfly solutions). These conventional packages are typically rigid and bulky and therefore not suited for emerging optical applications such as unobtrusive sensing systems, which require flexible and thin optoelectronic components. Moreover, limiting the dimensions of the interrogation system also limits the influence of this interrogation system on the device under test (for example a composite laminate). The driving and read-out interrogation units can even be fully embedded in the device under test, consequently avoiding delicate input and output transitions of sensing fibers (traveling from the device under test to an optical interrogation system).

### 4.1. Ultra Thin Flexible Optoelectronic Package

**Figure 8.**
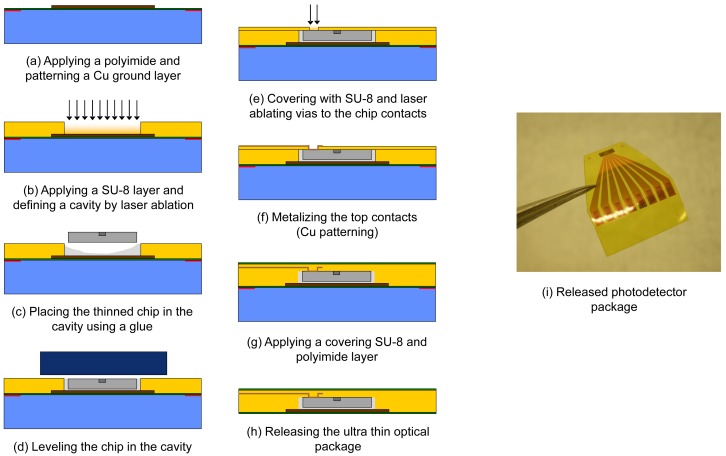
Chip package integration process flow.

Commercially available optoelectronic chips are therefore thinned down to 20 μm through a dedicated lapping and polishing process. The fabrication of the ultra thin optical chip package itself starts on a rigid temporary glass carrier on which a thin and mechanically strong polyimide layer (PI-2525, HD Microsystems) is spin-coated. A copper heat sink layer, which can also function as a back contacting layer, is sputtered and structured on top (wet etch process, Figure 8(a)). An SU-8 embedding layer is spin-coated on top and, using a CO_2_ and excimer laser, a chip embedding cavity is ablated (Figure 8(b)) to mount, level and fix an optoelectronic chip (Figure 8(c,d)). A thin polymer covering layer is applied on top of the chip and microvias are drilled using an excimer laser to access the chip contact pads (Figure 8(e)). A top contacting 1 μm Cu layer is sputtered and structured (Figure 8(f)) and again a thin polymer layer is spin-coated on top (Figure 8(g)). The final processing layer consists of a thin polyimide layer and the flexible optoelectronic package with a total thickness of 40 μm is now ready for release (Figure 8(h)). This generally described embedding process can also be used to embed multimode VCSELs in integrated flexible optical interconnects [16], to combine multimode VCSELs and photodetectors with a deformable transducer layer creating a flexible shear sensor [17] or to fabricate a highly accurate integrated incremental pressure sensor based on self-mixing interferometry in laser diodes [18]. In this paper however, we apply this process on single-mode VCSELs for fiber sensing purposes. Next to the preservation of single-mode behavior after chip thinning, polishing and embedding, efficient fiber coupling of the VCSELs is also an important prerequisite for this application. In contrast to the multimode components, the single-mode VCSELs we are using have cathode contact pads on the backside surface of the chip. Therefore, back contact layers need to be reapplied using an AuGeNiAu sputtering and evaporation process ending with a thermal annealing step. An example of an embedded photodetector array chip (available as bare chip from Enablence) is shown in Figure 8(i).

### 4.2. Integrated Fiber Coupling

The next step towards a fully embedded fiber sensing system is coupling the optoelectronic package to a fiber preserving the ultra thin characteristics and the associated advantages of the optical package. Fiber coupling is consequently performed through 45° micromirrors resulting in a planar fiber pigtailed package.

**Figure 9.**
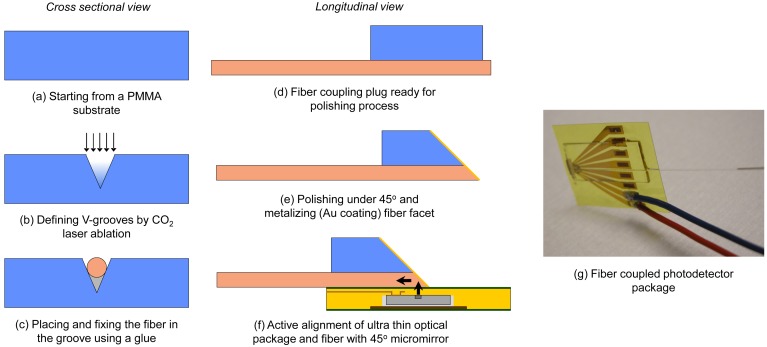
Integrated coupling process flow.

The fabrication of the fiber coupling plug starts on a 500 μm PMMA substrate (Figure 9(a)). V-grooves are ablated using a CO_2_ laser in order to position the coupling fiber at the edge of the PMMA plug (Figure 9(b)). A fiber (typically multimode silica) is subsequently aligned and fixed inside the groove (Figure 9(c)). Different grinding and polishing steps are performed under a controlled angle while monitoring surface roughness and fiber facet integrity after every step (Figure 9(d,e)). A high quality (average surface roughness of 23 nm on a 50 × 50 μm^2^ area) fiber coupling facet is obtained and a reflecting layer is applied by evaporating a thin layer of gold. The coupling plug is to be mounted directly on top of the optoelectronic package (Figure 9(f)) resulting in a flexible pigtailed optoelectronic package with a thickness well below 1 mm. By applying the reflecting mirror directly on the coupling fiber, the free space optical path length is minimized. To further maximize the fiber coupled power, the coupling fiber typically has a 50 μm core diameter and the alignment process of the coupling plug is performed actively. Depending on the application, the ultra thin optoelectronic packages (Section 4.1) can also be coupled to other light guiding structures. A process technology to couple embedded VCSELs and photodetectors to polymer waveguides or fiber arrays for flexible or even stretchable optical interconnects is described in [19]. An example of a fiber coupled photodetector array chip is shown in Figure 8(g).

As already mentioned in Section 3.3, not only the optoelectronic driving units but also the fiber sensors themselves can be embedded in polymer sheets with a typical thickness of 1 mm. Fiber embedding techniques are discussed in more detail in [20,21]. The final result is then a polymer foil with both integrated driving optoelectronics and optical sensor elements.

## 5. Fully Embedded System: Experimental Results and Discussion

The integration and coupling techniques discussed in the previous section (Figures 8 and 9) were applied on VCSEL and photodetector components to build a fully embedded fiber sensing system. This fully embedded system included the optoelectronic components as shown in Figure 2. Modulation mode was used to obtain full spectral reconstruction of the fiber sensor. Note that only relevant wavelength ranges are shown in the characterization graphs.

### 5.1. Temperature and Axial Strain Characterization

A fully embedded sensor system, consisting of a fiber coupled integrated single-mode VCSEL (available as bare chip from ULM Photonics), a fiber coupled integrated photodetector (available as bare chip from Enablence) and a silica fiber sensor with Bragg grating at 855 nm, was characterized under varying temperature and axial strain. To interrogate the fiber sensor, the electrical current through the VCSEL was driven using an analog sawtooth signal between 4 mA and 8 mA at 100 Hz. The fiber sensor was read out in transmission mode and the photodetector current was amplified and sampled at 100 kHz yielding 1,000 datapoints each tuning cycle of 10 ms.

**Figure 10.**
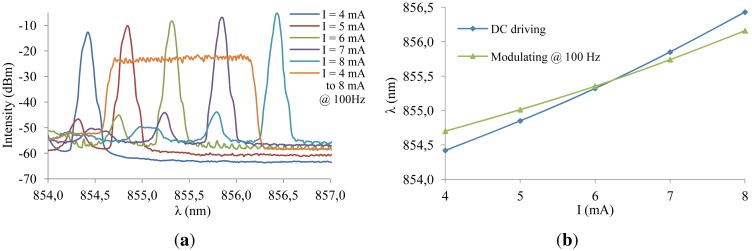
Dynamic wavelength range of the VCSEL. (**a**) Spectral intensity of the VCSEL measured using an Optical Spectrum Analyzer; (**b**) Wavelength tuning characteristics of the VCSEL.

**Figure 11.**
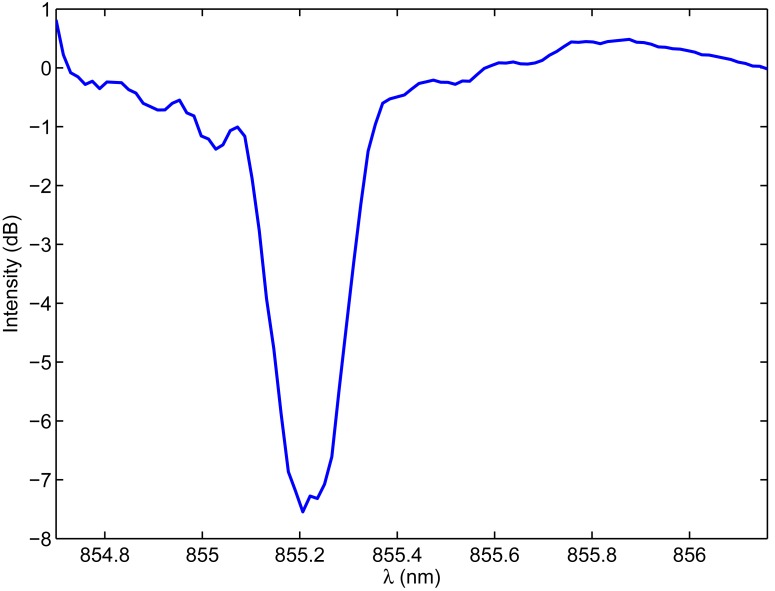
Full spectral FBG reconstruction using integrated VCSEL and photodetector.

Figure 10 shows the electro-thermal tuning effect of the integrated VCSEL as a function of different DC driving currents and the sawtooth signal at 100 Hz. The heating effect and the corresponding dynamic wavelength range is limited to 1.46 nm when using the sawtooth driving signal *versus* 2.01 nm when using the DC driving mode. A theoretical spectral resolution of 1.5 pm and a response time of 10 ms are consequently obtained. The accuracy of the interrogation system is limited by the VCSEL bandwidth. Figure 10(a) also indicates that single-mode behavior of the VCSEL is preserved for higher driving currents. Figure 11 shows the dynamic reconstruction of the 855 nm FBG using the integrated and fiber pigtailed VCSEL and photodetector, perfectly corresponding to the initial characterization results from Figure 1.

**Figure 12.**
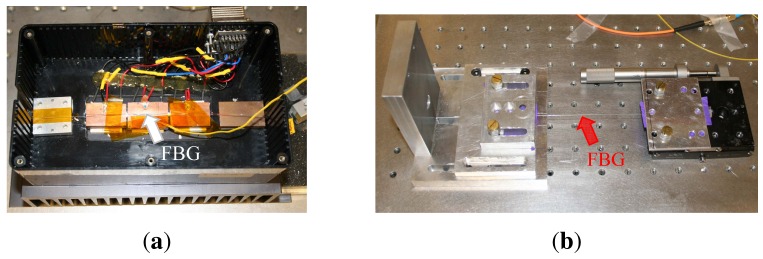
Fully embedded system tests. (**a**) Temperature characterization set-up; (**b**) Axial strain characterization set-up.

Temperature tests were carried out using a thermoelectric cooler (TEC) set-up on which the FBG was positioned next to the heating elements (Figure 12(a)). The axial strain tests were performed on a fiber clamping set-up with microscrews to accurately control the applied displacement (Figure 12(b)). Before the actual experiments, a calibration measurement was performed to obtain relative (referenced) spectral measurements.

**Figure 13.**
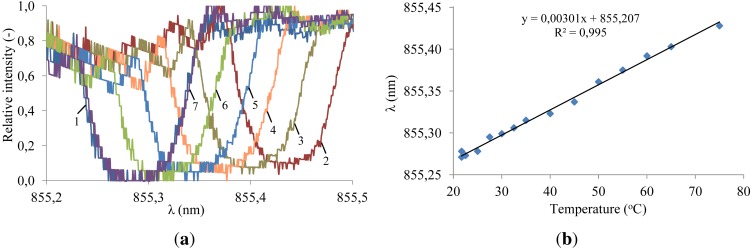
Temperature characterization of the fully embedded system. (**a**) 1: 21.7 °C (RT), 2: 75 °C, 3: 65 °C, 4: 55 °C, 5: 45 °C, 6: 35 °C, 7: 25 °C. Measured transmission spectra using the integrated VCSEL and photodetector system; (**b**) Wavelength extremum *versus* temperature.

Temperature characterization was carried out by first heating up the fiber from room temperature (Figure 13(a), measurement 1) to 75 °C (Figure 13(a), measurement 2) and subsequently cooling it down. A limited set of spectral measurements is shown in Figure 13(a). The central wavelength for all the measurements is plotted *versus* temperature in Figure 13(b). A linear temperature response is obtained yielding a sensitivity of $3.0\;\frac{\text{pm}}{{}^{\circ}\text{C}}$. The slope of this curve is deviating from the theoretical sensitivity of $5.7\;\frac{\text{pm}}{{}^{\circ}\text{C}}$ for 855 nm silica fiber Bragg gratings [22]. This difference can be explained by the heat loss during the heat transfer from the TEC to the fiber.

**Figure 14.**
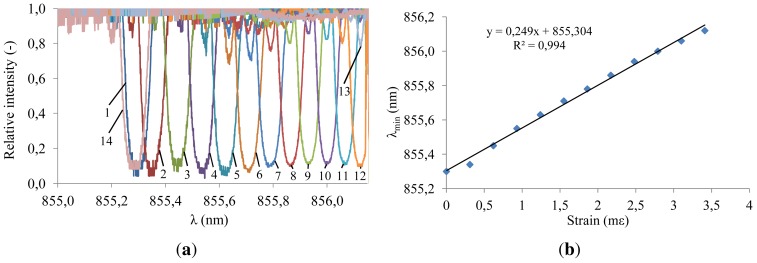
Strain characterization of the fully embedded system. (**a**) 1: 0 m*∊*, 2: 0.31 m*∊*, 3: 0.62 m*∊*, 4: 0.93 m*∊*, 5: 1.24 m*∊*, 6: 1.55 m*∊*, 7: 1.86 m*∊*, 8: 2.17 m*∊*, 9: 2.48 m*∊*, 10: 2.79 m*∊*, 11: 3.10 m*∊*, 12: 3.41 m*∊*, 13: 3.72 m*∊*, 14: 0 m*∊*. Measured transmission spectra using the integrated VCSEL and photodetector system; (**b**) Wavelength extremum *versus* axial strain.

Axial strain characterization tests were performed by applying different displacements on one end of the fiber and monitoring the corresponding spectra (Figure 14(a)). The central wavelength for all the measurements is plotted *versus* strain in Figure 14(b). A linear axial strain response is achieved yielding a sensitivity of $0.25\;\frac{\text{nm}}{\text{m}∊}$. This slope is lower than the theoretical sensitivity of $0.67\;\frac{\text{nm}}{\text{m}∊}$ for 855 nm silica fiber Bragg gratings [22]. The difference can be explained by partial slippage of the fiber in the fiber clamping set-up and partial slippage of the fiber in the acrylate fiber coating.

### 5.2. Dynamic Characterization

**Figure 15.**
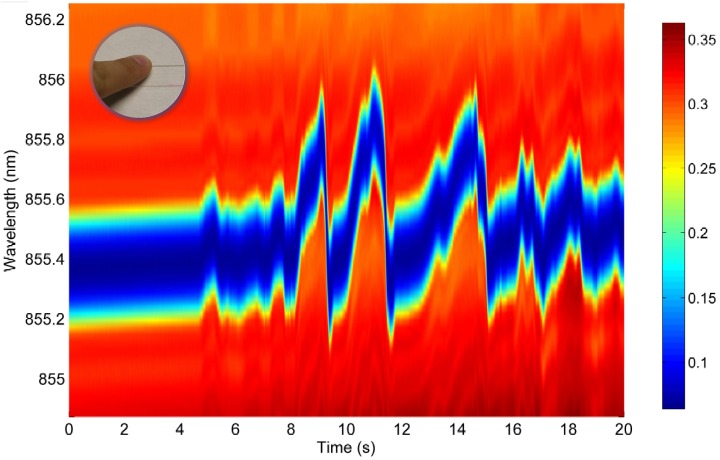
Tactile sensing demonstration: relative wavelength intensity variations when touching the embedded fiber sensor.

**Figure 16.**
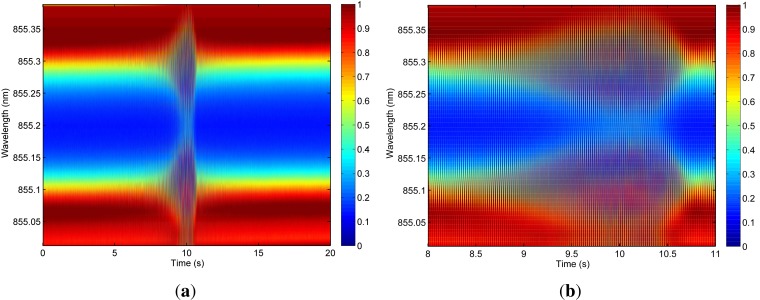
Electrodynamic shaker characterization of the fully embedded system (relative wavelength intensities). (**a**) Full spectral reconstruction of the resonance effect; (**b**) Zoomed view of the full spectral reconstruction.

**Figure 17.**
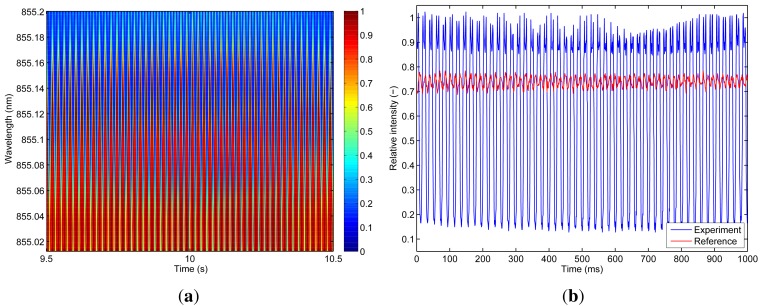
Detailed analysis of the electrodynamic shaker characterization (relative wavelength intensities). (**a**) Detailed view of the full spectral reconstruction; (**b**) Time domain analysis (intensity at a fixed wavelength of 855.104 nm) during resonance.

Dynamic characterization tests were performed to prove the feasibility of fast full spectral reconstruction measurements, not limited to a fixed monitoring wavelength. Therefore, the system was operated using a sawtooth signal of 1 kHz and driving currents between 4 mA and 8 mA. The corresponding wavelength range is limited to 1.39 nm in this case and a response time of 1 ms is consequently obtained. Data acquisition rates are varying between 100 kHz and 10 MHz, depending on the required measurement detail and associated resolution. The fiber sensor is embedded in an artificial sensing skin as discussed in [20,21]. Firstly and by means of demonstration, the operation of the sensing system was qualitatively proven by manually touching and releasing the fiber sensing skin and dynamically monitoring the system (*modulation mode* as schematically shown in Figure 3) resulting in accurate tactile sensing measurements (Figure 15). Furthermore, an electrodynamic shaker test was set up to dynamically characterize the system in a repeatable and controllable way. The fiber sensor was fixed on a metal resonating plate with a resonance frequency of 50 Hz. A logarithmic frequency sweep between 20 Hz and 300 Hz during 60 s was applied on the shaker set-up. Part of the resulting dynamic measurement (wavelength resolution of 4 pm) is shown in Figure 16(a) and more detailed views are depicted in Figures 16(b) and 17(a). In Figure 17(b), a time-domain plot (at a fixed wavelength, *λ* = 855.104 nm), focusing on the lower edge of the grating response, is depicting the periodic signal without shaking (labeled “Reference”) and during resonance (labeled “Experiment”). Without resonance, there is partial overlap of the VCSEL emitting power spectrum and the grating filter characteristic, transmitting about 75% of the optical power. During resonance, the Bragg grating filter characteristic overlap is completely eliminated when reaching a 100% relative transmitted power intensity. After analyzing this graph, a frequency of 50 Hz is obtained confirming that the spectral information can indeed be reconstructed correctly.

## 6. Conclusions

We have presented a new fiber sensor interrogation system which can be fabricated ultra small and fully embedded in a thin polymer stack. The sensing elements are manufactured using mature and commercially available Bragg grating inscription technology. Driving and read-out electronics and optoelectronics were combined with an Android based acquisition unit resulting in a portable and compact sensing system. Furthermore, technology was provided to take the integration a step further and embed the optoelectronics on a chip level to obtain a wearable system with a thickness below 1 mm. A broad range of new applications, including tactile sensing, structural health monitoring and biomedical systems, can be targeted with this unique sensing concept.

The interrogation technique is based on an intelligent VCSEL-photodiode combination. Two interrogation modes are available: constant current mode and modulation mode. In constant current mode, the transmitted optical power is monitored at a fixed wavelength with a maximum acquisition rate of 5 kHz. Using the modulation mode offers full spectral reconstruction. The corresponding wavelength resolution is depending on the driving frequency and data acquisition rate, and demonstrated at, but not limited to, 1.5 pm and 4 pm. The wavelength accuracy is limited by the VCSEL bandwidth and the associated response time varies between 1 ms and 10 ms.
